# Superficial Temporal Artery Perforator Flap: Indications, Surgical Outcomes, and Donor Site Morbidity

**DOI:** 10.3390/dj8040117

**Published:** 2020-10-12

**Authors:** Raffaele Rauso, Giovanni Francesco Nicoletti, Enrico Sesenna, Carmelo Lo Faro, Fabrizio Chirico, Romolo Fragola, Giorgio Lo Giudice, Gianpaolo Tartaro

**Affiliations:** 1 Oral and Maxillofacial Surgery Unit, Multidisciplinary Department of Medical-Surgical and Dental Specialities, University of Campania “Luigi Vanvitelli”, 80138 Naples, Italy; raffaele.rauso@unicampania.it (R.R.); romolofragola@gmail.com (R.F.); gianpaolo.tartaro@unicampania.it (G.T.); 2 Plastic Surgery Unit, Multidisciplinary Department of Medical and Dental Specialties, AOU University of Campania “Luigi Vanvitelli”, 80138 Naples, Italy; giovannifrancesco.nicoletti@unicampania.it; 3 Head and Neck Department, Maxillo-Facial Surgery Division, University Hospital of Parma, 43126 Parma, Italy; enrico.sesenna@unipr.it; 4 Maxillofacial Surgery Unit, Department of Neurosciences, Reproductive and Odontostomatological Sciences, University of Naples “Federico II”, 80138 Naples, Italy; fabriziochirico@hotmail.com (F.C.) Giorgio.logiudice@gmail.com (G.L.G.)

**Keywords:** superficial temporal artery island flap, intraoral defect, free flap combination, reconstructive surgical procedures, donor site morbidity

## Abstract

The aim of this retrospective case series was to discuss indications, surgical outcomes, and donor site morbidity in the use of superficial temporal artery perforator (STAP) flaps in intra-oral or extra-oral facial reconstruction. This study involved 9 patients treated with a STAP flap at the Maxillo-Facial Surgery Unit of the University of Campania “Luigi Vanvitelli”, Naples. A STAP flap was used alone or in combination with other local flaps, for the coverage of facial soft tissue defects, after the resection of craniofacial malignant tumors (*n* = 7) or as a salvage flap, in partial or total microvascular flap loss (*n* = 2). The STAP flap was proven to be a valuable surgical option despite it not being frequently used in facial soft tissue reconstruction nor was it chosen as the first surgical option in patients under 70 year’s old. Donor site morbidity is one of the major reasons why this flap is uncommon. Appropriate patient selection, surgical plan, and post-surgical touch-ups should be performed in order to reduce donor site scar morbidity.

## 1. Introduction

The first goal in facial reconstruction is anatomical repair while respecting the aesthetic units [1]. Facial reconstructive procedures are extremely heterogenous and can be tailored on the basis of the location and the size of the defect [2]. Currently, several surgical techniques for intra-oral and extra- oral facial defects can be performed, including free flaps [3], local and rotational flaps [4], pedicle flaps [5], and skin grafts [6], although scar quality and donor site morbidity must be carefully evaluated in the pre-operative planning.

Free flaps such as free radial forearm or anterolateral thigh perforator flap, when possible, always represent the first choice [7]. However, poor clinical condition, depleted vessels necks, previous radiotherapy, and elderly patients could represent relative contraindication to microsurgery [7].

The superficial temporal artery perforator (STAP) flap might represent a versatile surgical option in such clinical scenarios.

The temporal region is an excellent donor site because of its rich vascular network and its multitude of tissues types, including skin, fascia, muscle, and the calvarial bone. For this reason, single or composite flaps can be harvested from this site, which are based on the superficial temporal artery, and the path of its branches, and are chosen according to the defect site and nature [8].

Some papers describe the STAP flap as a good option in restoring several anatomic areas such as forehead, eyebrow, eyelid, cheek, nose, and also for intra-oral reconstructions [9,10,11,12,13,14]. The anatomy of the pedicle is reliable and constant and the wide rotation angle of the pedicle (up to 180 degree), allows large defect reconstruction (4–15 cm) [12].

In our opinion, donor site morbidity, including any subsequent alopecia and scarring, is one of the major reasons why the use of the STAP flap is still uncommon in practice.

Up to 3–4 cm of donor area defect can be closed primarily [15], although brow asymmetry can be detected postoperatively; moreover, bigger donor sites usually require a split thickness skin graft to resurface the area, resulting in poor aesthetic results secondary to skin color mismatch, and a thickness defect compared to the surrounding tissue or alopecia [16]. Surgical and non-surgical procedures can be further performed to reduce donor site morbidity.

The aim of this study was to discuss indications, surgical outcomes, and donor site morbidity in the use of STAP flap in intra-oral and extra-oral facial reconstruction.

## 2. Materials and Methods

This study was conducted retrospectively by evaluating the medical report of patients treated at the Maxillo-Facial Surgery Unit of the University of Campania “Luigi Vanvitelli”, Naples, between May 2018 and January 2020. A total of 9 patients (6M, 3F; 62–87 years) were recruited. A primary indication for STAP flap was given only in the case of defects involving the ear (Case 2), in an old patient with poor clinical conditions (Case 7), and in one case who had a small defect where a direct skin closure of the donor site was achievable without significant eyebrow asymmetry (Case 8). In 3 patients, this flap was used because of neck depleted vessels (Case 3, Case 6, and Case 9), in 2 patients it was used as a salvage procedure of partial microsurgical flap failure (Case 1 and Case 5), and in 1 patient it was used as a second flap, associated with a radial forearm free flap, following maxillary cancer demolition (Case 4).

The study followed the Declaration of Helsinki on medical protocol and ethics and due to the retrospective nature of this study, it was granted an exemption in writing by the University “Luigi Vanvitelli Naples Institutional Review Board.”

### Surgical Technique

Superficial temporal artery (STA) is the superficial branch of the external carotid artery system. The anatomical position of superficial temporal artery is relatively constant between patients and the pedicle is also easily exposed. STA usually has 2 main branches just above the ear, namely the posterior (parietal) and anterior (frontal). The anterior branch divides into 2 main terminal branches—frontal and parietal. Each of these branches allows the surgeon to harvest a different flap—the parietal branch is used when hair bearing is required for the reconstruction (eyebrow, mustache, etc.); the frontal one is preferred when hair is not required for the reconstruction. Usually, the main branch of the superficial temporal artery can be identified by digital palpation 1-cm in front of and 1-cm above the tragus, that said, it is also useful to identify and follow it along its main course, with a handle doppler.

An explorative skin incision, of about 3 cm, is usually performed, as in face-lift procedures, hidden in the pre-auricular fold. Once the superficial temporal pedicle is identified, skin incision can be performed cranially across the hair line and the pedicle can be followed in a free style technique.

When the parietal branch is not required for the flap, it has to be ligated as it emerges from the main pedicle, usually 1 cm above the ear. The vascular pedicle is followed up to the length required (a maximum of 15 cm) in order to have a wide arc of rotation, without tension or torsion of the pedicle itself, in order to close the defect.

Since the superficial temporal artery and vein do not run closely together along the entire course of the pedicle, the STAP flap has a well-defined arterial blood supply but frequently no single venous outflow. Its venous outflow might run through the peri-arterial tissue and the temporal artery pedicle should not be skeletonized to its core. Consequently, the flap might be observed in a varying blue tone in the first post-operative days.

Once the surgeon reaches the required pedicle length, a myo-cutaneous flap can be harvested, distally to proximally, sparing the periosteum layer, in order to have a good graft healing. The flap is usually designed leaving the pedicle at the center of it. Along the pedicle length, only a few collateral branches can be identified and ligated, some more branches carrying blood to the ear and to TMJ can be identified at the emerging point of STA; in case some more pedicle length is required, these branches have to be ligated. A wide subcutaneous tunnel between the flap and the recipient site is then performed and the flap is inset and adapted into the defect site.

## 3. Results

In all cases, the flap healed without major complication, although one patient (Case 4) who was treated with a synthetic acellular dermal regeneration template (Integra Lifesciences, Plainsboro, NJ, USA) before skin grafting, died 3 weeks after surgery, due to cardiopulmonary distress, with the template still in site. Direct closure of the donor site was achieved in 2 patients (Case 5 and Case 8), in 6 patients (Case 1, Case 2, Case 3, Case 6, Case 7, and Case 9), the donor site was skin grafted, and in 1 more patient (Case 4), a synthetic acellular dermal regeneration template (Integra Lifesciences, Plainsboro, NJ, USA) was temporarily placed.

**Figure 1:**
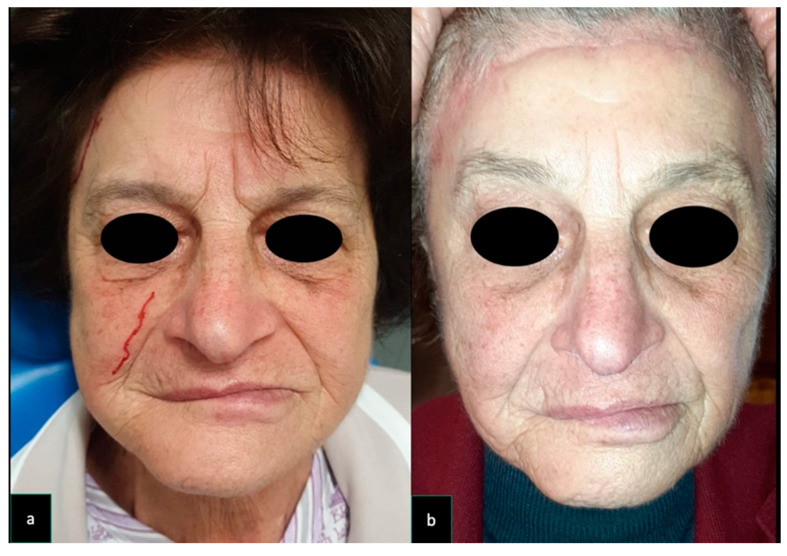
Case 6. A 79-year old woman underwent surgical excision for a recurrent left tuber maxillae squamous cell carcinoma. The patient had previously undergone a homolateral neck dissection and radiation therapy resulting in a depleted vessel neck. For this reason, a pedicle superficial temporal artery flap (9 × 9 cm) was raised and used to reconstruct the oral defect. The flap donor site was repaired with a skin graft and to reduce donor site scar morbidity, a forehead lift was implemented. (**a**) Pre-operative facial appearance of the patient. (**b**) Post-operative facial appearance of the patient. In order to reduce donor site morbidity and to achieve a symmetrical post-operative appearance of the forehead, a forehead lifting was performed.

**Figure 2:**
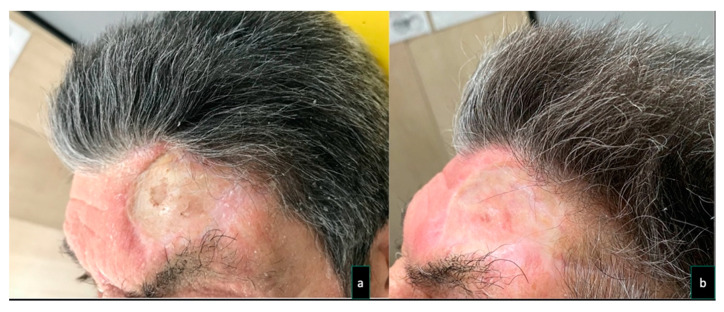
Case 3. A 74 -year old man underwent surgical excision for a recurrent left tuber maxillae squamous cell carcinoma. The patient previously underwent a homolateral neck dissection and radiation therapy, resulting in a depleted vessel neck. For these reasons, a pedicle superficial temporal artery flap (7 × 7 cm) was raised and used to reconstruct the oral defect. The flap donor site was repaired with a skin graft. Five sessions of a 20 mg/mL cross-linked hyaluronic acid (HA) filler (Hyamira Basic, Nyuma pharma^®^, Arona, Italy) was injected into the grafted area. About 0.2 mL of HA per session was injected over a total of 5 sessions, spaced 30 days apart. After the HA was injected into the graft site, a better skin quality with a more elastic tissue, and a less depressed appearance of the area were noticed. (**a**) The concave appearance of the grafted site was improved, following several sessions of cross-linked HA injections (**b**).

To reduce donor site scar morbidity, in one patient (Case 6) forehead lift was associated with STAP flap harvesting (Figure 1), and in another patient (Case 3) repeated hyaluronic acid injections were performed to reduce the concave appearance of grafted site (Figure 2). The patients were followed up for a minimum of 6 months (range 6*–*18 months).

**Figure 3:**
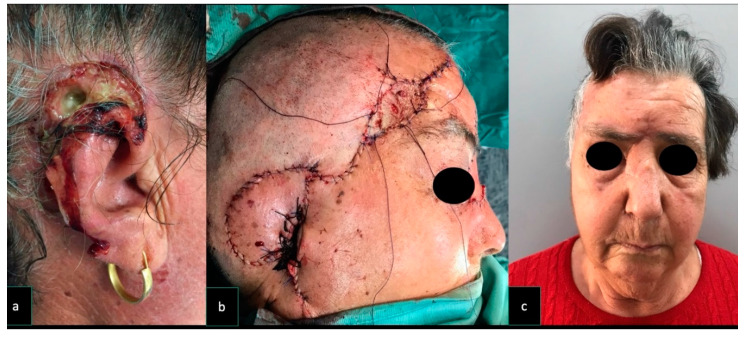
Case 2. A 76-year old woman underwent excisions for a squamous cell carcinoma involving the external ear and mastoid area A pedicle superficial temporal artery flap (9 × 7 cm) was raised and used to reconstruct the ear defect. The flap donor site was repaired with a skin graft and a local advancement flap was harvested to reduce the skin graft size. At 6-months follow-up, the patient was pleased with the aesthetic result and was disease free. (**a**) Pre-operative appearance of a cancer involving external ear and mastoid area. (**b**) The reconstruction performed with STAP flap; the donor site was skin grafted. (**c**) Post-operative symmetric appearance of the brows at rest.

In every case, symmetry of the eyebrows was achieved (Figure 3). Patient’s demographic data, surgery motivation, donor site morbidity, and the size of the flap used are reported in Table 1.

## 4. Discussion

The superficial temporal artery pedicled flap was first described by Dunhan in 1893 and was used to repair various facial defects [16].

Scaglioni et al. in 2015 [12], and Elbanoby et al. in 2016 [17], in anatomy-based clinical papers, described STAP flap harvesting with several innovations compared to the conventional technique.

The parietal branch of the STA offers a good operative choice if hair is needed for a beard, mustaches, or eyelid reconstruction, and usually the donor site is grafted and hidden by the surrounding hair [15,18].

**Figure 4:**
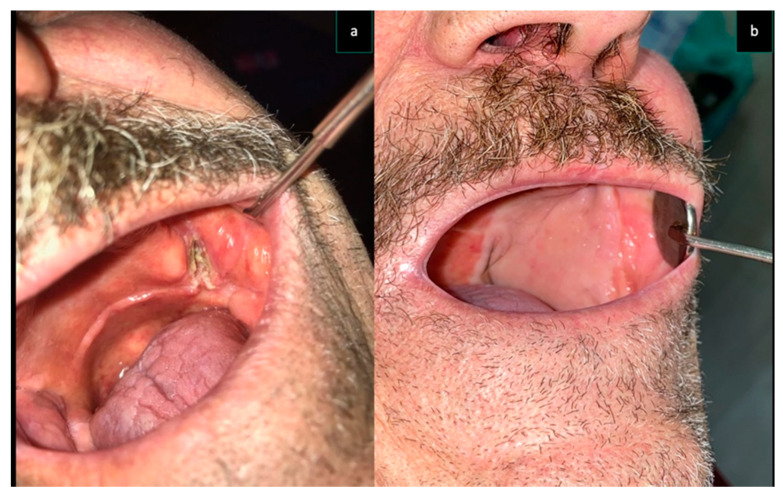
Same patient as Figure 2 (Case 3) (**a**) Intra-oral aspect of the cancer involving left tuber maxillae and cheek. The reconstruction performed with STAP flap (**b**).

**Figure 5:**
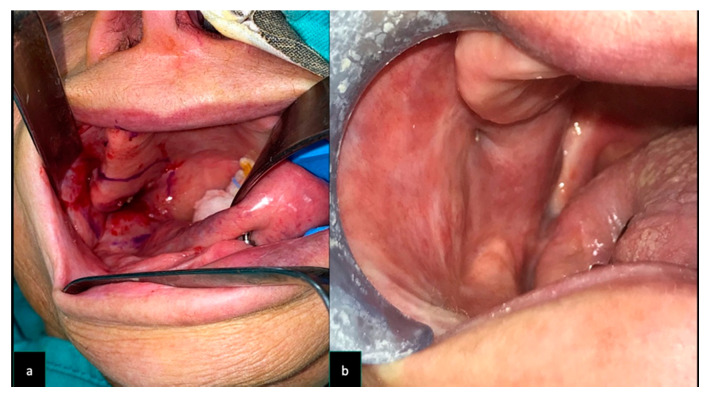
Same patient as Figure 1 (Case 6) (**a**) Intra-oral aspect of the cancer involving right tuber maxillae and cheek. (**b**) The reconstruction performed with STAP flap.

In the present study, we only used the frontal branch of STA because hairless flaps were needed for reconstruction. (Figure 4 and Figure 5).

Donor site morbidity, including alopecia, dysfunctional and unaesthetic scarring, are amongst the main reasons why the use of STAP flap is still uncommon in practice, despite several advantages like providing a thick and long pedicle with an average external diameter of the superficial temporal artery more than 2.8 mm, a consistent anatomical position, and ease of its harvest.

Similarly, in our experience, a STAP flap is not the first choice in patients under 70 year’s old, especially considering the need for forehead sagging skin that is used to reduce donor site morbidity. Our youngest patient presented in this case series was a 62 year’ old and a STAP flap was performed to overcome a non-preoperatively planned excision of the skin during maxillary cancer removal. This case displayed the versatility and reliability of this flap “in case of need”. In this patient, we applied a synthetic acellular dermal regeneration template (Integra Lifesciences, Plainsboro, NJ, USA) onto the donor site, in order to achieve proliferation of the underlining tissue and had a three-dimensional reconstruction, only grafting the site few weeks later to the template application.

Patients who were over 70-years-old usually had enough lax tissue above the forehead and the use of local advancements flaps allowed the surgeon to have a direct wound closure or reduction of the site to graft.

A major concern regarding the STAP flap, when harvested above its frontal branch, was the asymmetry of the eyebrows occurring after direct closure of donor site [19], or the appearance and the size of donor site when the skin was grafted. Elbanoby et al. in 2018 [20] reported their experience with STAP flap and classified the application of superficial temporal artery flaps to reconstruct a wide range of facial defects, and organized these guidelines as an algorithmic approach. In their paper, Elbanoby et al. [20] underlined how the limitations of the use of the island superficial temporal artery flap could result in donor site morbidity, and the need for second-stage refinements of the flap.

According to this algorithmic approach, we found this flap to be reliable in restoring both intra-oral and extra-oral defects and some considerations arose while reducing donor site morbidity.

In cases where the pedicle length was between 5 and 7 cm and a maximum 5 × 5 cm sized flap was needed, direct donor site closure could be achieved by performing a rotational advancement of the lower flap, moving it from central to lateral. When bigger flaps, or longer pedicles, are required, especially for intra-oral reconstructions, skin grafting is mandatory to avoid post-operative complications regarding asymmetry of the eyebrows. Once the flap is harvested, a rotational advancement of the lower flap, moving it from central to lateral, has to be performed, in order to reduce the size of the graft. In these cases, the area to be grafted should fall just above the hairline in the temporal region, easily hidden by the hair of the patient. A useful step to conduct once the flap is harvested, is to perform a frontal–temporal lift of the contralateral side when larger flaps are required (9 × 9 cm or more). We performed this technique in Case 6, harvesting a 9 × 9 cm flap, grafting the donor site with a 3 × 1 cm skin graft, and achieving good eyebrows symmetry, post operatively. A STAP flap is also a reliable option after neck dissections, in the so-called vessels depleted necks. Local facial rotational/advancement flaps are mainly based on the facial artery pedicle, often ligated during neck dissection and, although a retrograde revascularization of the facial artery can be achieved, the superficial temporal artery seems to be a more reliable choice.

Some authors consider post-operative eyebrow asymmetries to be a direct consequence of STAP flaps. Ozdemir et al. [21] argued that the STAP flap causes eyebrow asymmetry and for this reason Z-plasty, transposition flap, or sling procedures should be performed to the opposite side.

We agree with these suggestions, although we prefer to overcome this issue with a careful preoperative plan and doing our best to reduce eyebrows asymmetry, while adjusting the technique intra-operatively.

Another issue regarding skin grafting is the appearance of the grafted area.

Placing a skin graft directly over the periosteum of the frontal area to allow it to achieve a convex appearance. To overcome this issue in one patient (Case 3), once surgical sites were completely healed, 3 months after surgery, we came up to the surgical sequela of the donor site with a non-surgical approach, performing sub and supra-periosteal injections of a 20mg/mL crosslinked HA filler (Hyamira BASIC, NYUMA PHARM s.r.l **^®^**., Arona, Italy). About 0.2 mL of HA per session was injected for a total of 5 sessions, spaced 30 days each. Nine months after the last injection, the grafted site showed a better skin quality, a more elastic tissue, and a less depressed appearance (Figure 2).

## 5. Conclusions

STAP flaps were proven to be a valuable flap, despite its uncommon use in facial soft tissue reconstruction. We believe that it is also a good alternative for patients who have not considered free flaps. In some patients who have significant comorbidities or specific contraindications to free tissue transfer, a STAP flap provides an excellent alternative for reconstructing the defects of the oral cavity. Although a larger series of cases is needed, STAP flaps might represent a versatile surgical option, also allowing a wide range of reconstructions of different facial subunits. Donor site morbidity is one of the major motivations why this flap is uncommon. An appropriate patient selection, surgical plan, and post-surgical touch-ups could be performed in order to reduce donor site scar morbidity.

## Figures and Tables

**Table 1:** Patient’s demographic data, motivation for surgery, donor site closure, and the size of STAP flap used. BCC = Basal Cell Carcinoma; HA = hyaluronic acid; RFFF = Radial Free Forearm Flap.

Patient	Demographics	Motivation to Surgery	Surgical Procedure Associated to Cover the Defect	Surgical Procedure Associated to Reduce Donor Site Morbidity	Clinical Condition Associated	Donor Site Closure	Further Refinements Procedure	Size of the Flap
Case 1	72 y.o. (M)	Microsurgical flap failure		Local advancement flap to reduce graft size	Hypertension	Skin graft		15 × 7 cm
Case 2	76 y.o. (F)	Ear squamous carcinoma		Local advancement flap to reduce graft size	Hypertension; diabetes	Skin graft		9 × 7 cm
Case 3	74 y.o. (M)	L tuber maxillae carcinoma		Local advancement flap to reduce graft size	Neck depleted vessel, previous RT; hypertension	Skin graft	HA injection on the grafted site; surgical eyebrow touch up	7 × 7 cm
Case 4	62 y.o. (M)	L maxillary cancer	RFFF for the internal lining		Hypertension; diabetes	Synthetic acellular dermal regeneration template		7 × 7 cm
Case 5	78 y.o. (M)	Partial microsurgical flap failure		Local advancement flap to have direct closure	Diabetes	Direct closure		5 × 5 cm
Case 6	79 y.o. (F)	R tuber maxillae carcinoma	Forehead lift		Neck depleted vessel, previous RT	Skin graft		9 × 9 cm
Case 7	87 y.o. (M)	R Cheek/nasal side wall/eyelid (skin) squamous cell carcinoma	Glabellar flap and upper eyelid flap	Local rotational flap to reduce graft size	Hypercolesterolemia	Skin graft		5 × 5 cm
Case 8	72 y.o. (M)	R Cheek BCC		Local advancement flap to have direct closure		Direct closure		4 × 4 cm
Case 9	82 y.o. (M)	R Cheek BCC		Local advancement flap to reduce graft size	Neck depleted vessel, previous RT	Skin graft		5 × 4 cm
